# Diagnosis and treatment of uterine artery pseudoaneurysm: Case series and literature review

**DOI:** 10.1097/MD.0000000000028093

**Published:** 2021-12-23

**Authors:** Tingting Wu, Beibei Lin, Kui Li, Jinying Ye, Ruijin Wu

**Affiliations:** a Department of Gynecology, Women's Hospital, Zhejiang University School of Medicine, Hangzhou, Zhejiang province, People's Republic of China; b Department of Radiology, Women's Hospital, Zhejiang University School of Medicine, Hangzhou, Zhejiang Province, People's Republic of China.

**Keywords:** computed tomography angiography, conservative management, rupture, transarterial embolization, uterine artery pseudoaneurysm

## Abstract

**Background:**

Uterine artery pseudoaneurysm (UAP) is a rare but potentially life-threatening cause of hemorrhage. Nonetheless, its knowledge could be insufficient among obstetricians, gynecologists, and radiologists. We aimed to clarify the clinical characteristics, management, and outcomes of UAP.

**Methods:**

We retrospectively analyzed nine female patients diagnosed with UAP at our institute between 2013 and 2020.

**Results:**

Seven cases presented with a history of traumatic surgery including cesarean section, dilation and curettage, laparoscopic myomectomy, and cervical conization. Two cases occurred after spontaneous vaginal delivery and second-trimester pregnancy termination. The main symptom was heavy/massive/prolonged vaginal bleeding. All patients were first evaluated by color Doppler ultrasonography and three cases were confirmed by magnetic resonance imaging. Severn patients underwent transarterial embolization (TAE) of the uterine arteries, and two were managed conservatively. All patients had good outcomes.

**Conclusions:**

UAP can develop after traumatic pelvic operations and non-traumatic delivery/abortion. It may be more common than previously considered. The risk of rupture may be correlated with multiple factors other than the mass size. TAE of the uterine artery could be an effective management strategy for ruptured UAP. However, some cases can resolve spontaneously without TAE, suggesting that conservative management can be employed in some women.

## 1 Introduction

Uterine artery pseudoaneurysm (UAP) is a complication of pelvic surgery. It is usually detected after the rupture of the pseudoaneurysm, thereby manifesting as massive hemorrhage. Histologically, UAPs often consist of only one layer of loose connective tissue, which differentiates them from true aneurysms consisting of a complete three-layered wall. Extraluminal turbulent blood flow can lead to enlargement of the UAP, making it susceptible to rupture and subsequent bleeding.

UAP can not only develop after various traumatic obstetric or gynecological procedures, but also occur secondary to non-traumatic delivery/abortion.^[1]^ Two types of UAPs have been reported: one that communicates with the uterine cavity and causes genital bleeding, while the other that has no communication and results in hematoma formation outside the uterus. The latter type can cause hydronephrosis.^[2]^ Lack of timely diagnosis of UAP can lead to incorrect management and poor outcomes. UAPs are traditionally treated by hysterectomy and arterial ligation, while transarterial embolization (TAE) has been widely used for the management of UAP in recent years.^[3]^ Herein, we describe nine cases of UAP treated at our institution over the past seven years and include a review of the literature. We find that the location and the pressure of the UAP mass may be more meaningful and some UAPs can spontaneously resolve even without TAE.

## 2 Materials and methods

### 2.1 Patients

We extracted the electronic medical records of all patients at the Women's Hospital, Zhejiang University School of Medicine, over the last ten years. Nine female patients (mean age: 34.78 ± 8.74 years; mean gravidity: 2.11 ± 1.05, mean parity: 0.89 ± 0.6) identified with UAP from August 2013 to October 2020were enrolled. Written informed consent was obtained from the patients for the publication of their information and images. This study (IRB-20200288-R) was approved by the Ethics Committee of Women's Hospital, Zhejiang University School of Medicine.

### 2.2 Diagnosis of UAP

UAP was diagnosed based on one of the following findings: (1) Color Doppler ultrasound findings of an intrauterine mass with swirling blood flow, with a to-and-fro or yin-and-yang pattern; (2) magnetic resonance imaging (MRI) revealing an enhanced, pseudoaneurysmal sac-like structure within the uterus; (3) computed tomography angiography (CTA) confirming the presence of UAP with a narrow connection with the parent artery (uterine artery).

### 2.3 Medical data collection

We retrieved the data regarding age, gravidity, parity, the presence or absence of hemorrhage, occurrence of UAP after traumatic or non-traumatic events, the interval and the onset of symptoms that between the events preceding UAP and the diagnosis of UAP, the diagnostic modality, angiographic findings and the involved arteries, the size of masses, and the line of management.

### 2.4 Statistical analyzes

IBM SPSS Statistics (version 23.0; IBM SPSS Statistics for Windows, IBM Corp., Armonk, NY) was used for statistical analyses and calculations. Statistical significance was set at *P* < 0.05. Variables were tested for normality using the Kolmogorov-Smirnov test. Since variables in the present study were not normally distributed, the results were expressed using the Mann-Whitney test.

## 3 Results

The detailed characteristics of the nine patients with UAP are shown in Table 1.

**Table 1:** Postoperative characteristics of patients withuterine artery pseudoaneurysm (n = 9).

Variable	Value
Previous abdominal surgery	
Cesarean section, n	3 (33.3%)
Laparoscopic myomectomy, n	2 (22.2%)
Dilatation and curettage, n	1 (11.1%)
Cervical conization, n	1 (11.1%)
Second-trimester pregnancy termination, n	1 (11.1%)
Spontaneous vaginal delivery, n	1 (11.1%)
Interval between previous surgery and symptom onset (days; median)	25.33 ± 30.27
Interval between symptom onset and diagnosis (days; median)	46 ± 28.12
Signs and symptoms	
Vaginal bleeding, n	8 (88.8%)
Bleeding more than 600 ml, n	4 (44.4%)
Persistent slight bleeding, n	2 (22.2%)
Intermittent slight bleeding, n	2 (22.2%)
Abdominal pain and fever, n	1 (11.1%)
Size of UAP masses in the ruptured group (cm; median)	2.45 ± 0.38
Size of UAP masses in the unruptured group (cm; median)	4.04 ± 2.7
Management procedure	
Embolization, n	7 (77.7%)
Conservative management, n	2 (22.2%)
Embolic agents	
Gelatin sponges (bilateralsides), n	2 (28.6%)
Metal coils (affected side) and gelatin sponges (opposite side), n	5 (71.4%)

UAP = uterine artery pseudoaneurysm.

**Figure 1:**
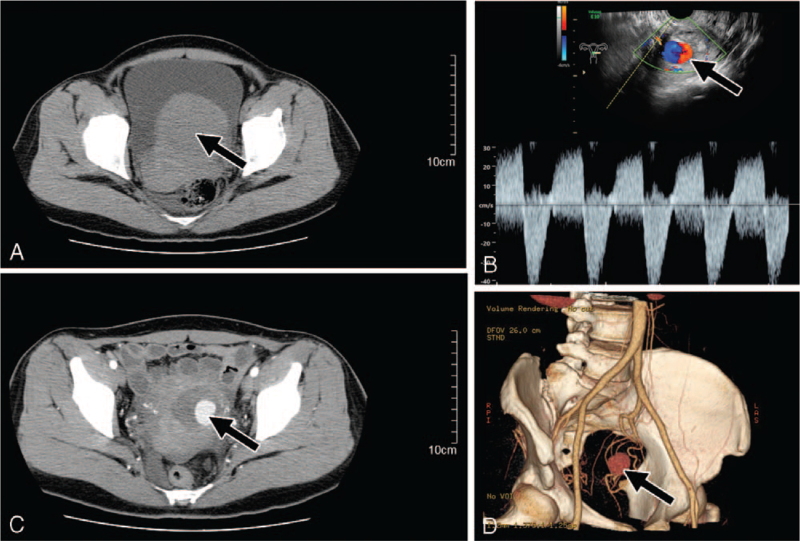
A uterine artery pseudoaneurysm developed after laparoscopic-assisted myomectomy in a 47-year-old patient. (A) Computed tomography showing 7.0 cm subserosalmyoma in the lower left anterior uterine wall (arrow); (B) Color Doppler ultrasonographic image showing swirling blood flow in the myomectomy scar (arrow) on postoperative day 19; (C) Magnetic resonance imaging revealing the enhanced pseudoaneurysmal sac-like structure within the uterus (arrow); (D) Three-dimensional computed tomography angiography showing the pseudoaneurysm originating from the left uterine artery (arrow).

All patients were initially evaluated using color Doppler ultrasonography. The presence of UAP was confirmed using CTA in six women and MRI in three women. TAE of the whole uterine artery was performed in seven patients with a confirmed diagnosis of UAP, in whom the feeding vessels originated from the uterine artery. Among these patients, one was a 47-year-old woman who underwent laparoscopic myomectomy for a subserosal fibroid in the left anterior isthmus of the uterus (Fig. 1A, arrow). Ultrasound imaging showed swirling blood flow in the myomectomy scar 19 days after surgery (Fig. 1B, arrow). MRI revealed a pseudoaneurysm that developed from the left uterine artery (Fig. 1C, arrow), that was confirmed by CTA (Fig. 1D, arrow).

Four patients suffered massive hemorrhage (>600 ml) in the late postoperative period (30–52days; mean interval: 36.5 days), and the other five patients experienced prolonged bleeding or abdominal pain and fever (20–90days; mean interval: 50.8 days). A comparison of the size of the UAP masses between the ruptured and unruptured groups is shown in Table 2.

**Table 2:** Comparison of the size of uterine artery pseudoaneurysm masses between the different groups.

	Ruptured group (n = 4)	Unruptured group (n = 5)	*U*	*P*
Size of UAP masses (cm; median)	2.45 ± 0.38	4.04 ± 2.7	5.5	>.05

UAP = uterine artery pseudoaneurysm.

There was no statistical difference between the two groups. Five patients were successfully treated with bilateral VTE. Among them, one patient who had external vaginal bleeding that markedly decreased after uterine balloon tamponade still underwent VTE. Conservative management was performed in two patients in whom the bleeding resolved spontaneously. The clinical characteristics of patients in the VTE and conservative management-groups are shown in Table 3.

**Table 3:** Clinical characteristics of patients in different groups.

Variable	Embolization group (n = 7)	Conservative management group (n = 2)
Previous events	traumatic operations	non-traumatic delivery/abortion
Ruptured	yes (57.1%);no (42.9%)	no
Symptom	massive hemorrhage (57.1%);slight bleeding (28.6%);abdominal pain and fever (14.3%)	slight bleeding
Size of UAP masses (cm; median)	3.57 ± 2.32	2.5 ± 0.99
Location of UAP	isthmus	myometrium

UAP = uterine artery pseudoaneurysm.

After a maximum follow-up of 8 months, all patients had normal menstruation patterns and no further vaginal bleeding. The patient who was conservatively managed is currently 20 weeks pregnant.

## 4 Discussion

The first case of UAP was reported in 1997.^[4]^ Its exact incidence has not yet been determined in women, and it has long been described as “very rare”. However, UAP can occur more frequently than previously expected(3–6/1000 deliveries),as reported by Baba et al.^[5]^ This suggests that many cases may be asymptomatic or resolve spontaneously, and therefore remain undiagnosed. Considering that UAP can cause life-threatening hemorrhage, it is important that obstetricians and gynecologists recognize and diagnose UAP early.

UAP can usually be diagnosed using color Doppler ultrasonography, CTA, and MRI. Ultrasound demonstrates turbulent arterial flow with a to-and-fro or yin-and-yang pattern that results from blood flow into the pseudoaneurysm, which is pathognomonic of pseudoaneurysm.^[6]^ The sensitivity and specificity of ultrasound can reach 95%.^[7]^ In the present case series, color Doppler ultrasound was the first choice of investigation. However, the limitation in the diagnostic ability of color Doppler ultrasonography is that it cannot reveal the precise vascular structure of the offending vessel from which the pseudoaneurysm has developed, so as to determinethe management. MRI can confirm the diagnosis and rule out other, more common vascular abnormalities such as arteriovenous malformations or fistulas.^[8]^ CTA can better identify the feeding vessels of UAP with minimal invasiveness and within a short period of time, and is a powerful diagnostic tool when abnormal vascular structures are identified by color Doppler ultrasonography.^[9]^ Active extravasation can be seen on angiography when UAPs rupture, and the bleeding site can be located for subsequent TAE. Recently, hysteroscopy has also been used for the diagnosis of UAP. Tsunoda et al. reported a case in which hysteroscopy was used to diagnose UAP that was difficult to differentiate from retained products of conception on ultrasound and CT.^[10]^

In the past, the main choice of treatment for UAP was open surgical management, including hysterectomy and ligation of uterine or internal iliac artery. Further, several case reports have mentioned the use of uterine balloon tamponade and laparoscopic surgery for the treatment of UAP.^[11,12]^ Treatment options have therefore evolved from surgery to TAE, which is safe and effective in recent years especially for patients who desire fertility preservation. The surgical approach may be more suitable in cases of acute and massive bleeding when there is no time for TAE. The choice of treatment may also depend on the specific resources available in each institution.^[13]^ In the present case, TAE was also set as the first choice of treatment. Except for the two self-resolving cases, the other seven cases were all treated with TAE. Among them, five were treated with permanent embolization materials, such as metal coils, whilegelatin sponge, which is absorbed after 2 to 3 weeks, was used on the opposite side. The other two cases treated with bilateral uterine artery embolization with gelatin sponge also had a good outcome. Cooper et al. reported that one case required repeat embolization of the contralateral side after unilateral uterine embolization because of the redistribution and redirection of blood.^[14]^ This indicates that bilateral uterine artery embolization may be more advantageous than unilateral embolization. Meanwhile, other feeding arteries, such as the ovarian artery, inferior epigastric artery, or middle acral artery should be examined when the bleeding does not stop.^[15]^ Pregnancy outcome would be a major concern for women with a strong desire for fertility. Studies have shown that pregnancy outcomes after TAE for UAP were favorable.^[16]^

UAP is usually associated with a prior traumatic surgical procedure, the most common of them beings cesarean section.^[17]^ They can also occur as a complication of myomectomy,^[18]^ hysterectomy,^[19]^ laparoscopic excision of deep endometriotic lesions,^[20]^ dilation and curettage,^[5]^ uterine cervical conization,^[21]^ and so on. UAPs can originate from a disruption of the continuity of the uterine arterial wall that is incidentally damaged during previous procedures. However, there have been some cases of UAP occurring after non-traumatic delivery or abortion.^[3]^ We also report two cases of UAPs developing after spontaneous vaginal delivery and second-trimester pregnancy termination. However, the two patients underwent hysteroscopic electrocision due to intrauterine adhesions before pregnancy. The mechanism of UAP occurrence after non-traumatic delivery/abortion is unclear; however, the shear force at the site during delivery may tear or injure the artery.^[22]^ Furthermore, we consider that muscle damage and subsequent infection caused by electrical cutting may be high-risk factors for UAP, which needs further study.

A few cases have reportedly resolved spontaneously with no necessity for specific treatment.^[5]^ In our study, there were two patients managed conservatively who developed no abnormalities over a short-term observation period. Compared with the embolization group, the two cases that occurred after non-traumatic delivery/abortion had slight vaginal bleeding and resolved spontaneously. We also found that the masses of UAP were at a certain distance from the isthmus and had smaller diameters. Takahashi et al. also reported three cases of spontaneous resolution and indicated that cases with few or no symptoms and small-diameter UAP may be candidates for spontaneous resolution.^[23]^ However, the masses in the four cases of ruptured UAP were not larger than those of the unruptured ones in our study. In addition, rupture can also cause the mass to become smaller, which indicates that the size of the masses may not be positively related to the risk of rupture. Baba et al. found that rupture of UAP may depend on the balance between the intra-UAP blood flow/pressure and UAP wall strength, and proposed that “absent diastolic flow” may predict spontaneous resolution of UAP.^[24]^ However, this was a case report. Whether the blood flow/pressure and UAP wall strength, rather than the size of UAP, can be a candidate for predicting UAP resolution needs to be studied further. Nonetheless, Baba's findings instruct us to consider routine inspections of the intra-UAP blood flow/pressure and UAP wall strength in the future.

The most common clinical manifestation associated with UAP is intermittent or persistent vaginal bleeding, while some cases are asymptomatic or present with fever and abdominal pain. The interval between prior history and symptom onset remains unknown, ranging from 6 to 140 days in case reports; one case of UAP presenting 10 years after cesarean section has been reported.^[7,22]^ In our cases, four patients suffered hemorrhage and hemodynamic instability. Fortunately, embolization was performed in a timely and successful manner to save patients’ lives and preserve fertility. No electronic records were available for the diagnosis of UAP before 2013. Thus, we speculated that gynecologists and radiologists may have insufficient knowledge of the disease, and the diagnosis may be missed. Therefore, it is important to disseminate knowledge about this dangerous entity among gynecologists and radiologists for the early detection and diagnosis of pseudoaneurysm before the manifestation of its catastrophic clinical symptoms.^[25]^ However, there are some limitations associated with this study. This was a retrospective study with a small sample size. In the next stage, prospective screening on patients will be carried out to further study on the rupture pattern of UAP.

## 5 Conclusions

In conclusion, UAP can be caused by various traumatic obstetric or gynecological procedures and non-traumatic delivery/abortion. Vaginal bleeding is the main symptom, while some cases are asymptomatic. CTA is regarded as the diagnostic gold standard, and TAE is required as the first-line of treatment. Our experience suggests that some cases develop after non-traumatic delivery/abortion with minor symptoms, and that small-diameter UAP can be managed conservatively because of spontaneous resolution. The location of the UAP mass may be more predictive of its prognosis than its size. Further studies with a larger sample size are necessary to better understand the natural history of UAP.

## Acknowledgments

The authors thank all of the patients who participated in the study.

## Author contributions

**Conceptualization:** Tingting Wu, WU Ruijin.

**Data curation:** Beibei Lin.

**Formal analysis:** Jinying Ye.

**Investigation:** Beibei Lin.

**Methodology:** Tingting Wu, Beibei Lin.

**Resources:** Tingting Wu, Kui Li.

**Writing – original draft:** Tingting Wu, Beibei Lin.

**Writing – review & editing:** WU Ruijin.
